# Cardiopulmonary Fitness and Physical Activity Among Children and Adolescents With Inherited Cardiac Disease

**DOI:** 10.1001/jamanetworkopen.2024.61795

**Published:** 2025-02-25

**Authors:** Luc Souilla, Oscar Werner, Helena Huguet, Arthur Gavotto, Marie Vincenti, Jean-Luc Pasquie, Gregoire De La Villeon, Sophie Guillaumont, Victor Pommier, Stefan Matecki, Alban-Elouen Baruteau, Constance Beyler, Christophe Delclaux, Isabelle Denjoy, Philippe Charron, Philippe Chevalier, Antoine Delinière, Mathieu Andrianoely, Lauriane Cornuault, Caroline Besnard-Neyraud, Frederic Sacher, Patricia Reant, Denis Mottet, Marie-Christine Picot, Pascal Amedro

**Affiliations:** 1 PhyMedExp, INSERM, CNRS, University of Montpellier, Montpellier, France; 2 Department of Pediatric and Congenital Cardiology, M3C Regional Reference Centre, Montpellier University Hospital, Montpellier, France; 3 Epidemiology and Clinical Research Department, Montpellier University Hospital, University of Montpellier, Montpellier, France; 4 Cardiology and Vascular Diseases Department, Regional Reference Centre for Inherited Cardiac Arrhythmia, Montpellier University Hospital, Montpellier, France; 5 Pediatric Cardiology and Rehabilitation Unit, Institut-Saint-Pierre, Palavas-Les-Flots, France; 6 Department of Pediatric and Congenital Cardiology, CHU La Reunion, University of La Reunion, Saint-Denis, France; 7 Nantes Université, CHU Nantes, Department of Pediatric Cardiology and Pediatric Cardiac Surgery, FHU PreciCare, Nantes, France; 8 Nantes Université, CHU Nantes, INSERM, CIC FEA 1413, CNRS, INSERM, l’institut du Thorax, INRAE, UMR 1280, PhAN, Nantes, France; 9 Pediatric Cardiology and Physiology Department, Robert Debre University Hospital, APHP, Paris, France; 10 Department of Cardiology, National Reference Centre for Inherited Cardiac Disease, APHP, Paris, France; 11 National Reference Centre for Inherited Arrhythmia of Lyon (CERA), Electrophysiology Unit, Hôpital Cardiologique Louis Pradel, Hospices Civils de Lyon, Bron, France; 12 Claude Bernard Lyon 1 University, MeLiS, CNRS UMR 5284, INSERM U1314, Institut NeuroMyoGène, Lyon, France; 13 Department of Pediatric and Adult Congenital Cardiology, National Reference Centre for Complex Congenital Heart Disease, Bordeaux University Hospital, Pessac, France; 14 IHU Liryc, Electrophysiology and Heart Modelling Institute, INSERM U1045, University of Bordeaux, Pessac, France; 15 National Reference Centre for Inherited Cardiac Arrhythmia, CMARY, ERN Guard-Heart, Bordeaux University Hospital, Pessac, France; 16 National Reference Centre for Inherited Cardiomyopathy, Bordeaux University Hospital, Pessac, France; 17 EuroMov Digital Health in Motion, University of Montpellier, IMT Mines Alès, Montpellier, France

## Abstract

**Importance:**

Historical restrictions on children with inherited cardiac arrhythmia or cardiomyopathy have been implemented to mitigate the potential risk of sudden death, but these limitations can be detrimental to overall health and cardiopulmonary fitness.

**Objectives:**

To evaluate cardiopulmonary fitness and physical activity among children with inherited cardiac disease and identify the factors associated with maximum oxygen uptake (V̇o_2_max) in this population.

**Design, Setting, and Participants:**

This cross-sectional, multicenter, prospective controlled study was conducted in 7 tertiary care expert centers for inherited cardiac disease in France from February 1, 2021, to June 20, 2023, with a 2-week follow-up. Participants included 100 children and adolescents aged 6 to 17 years with inherited cardiac arrhythmia or cardiomyopathy who were compared with 107 sex- and age-matched controls.

**Main Outcomes and Measures:**

Maximum oxygen uptake was assessed using cardiopulmonary exercise testing, and results were expressed using pediatric reference *z* score values. The main determinants of V̇o_2_max included clinical (New York Heart Association [NYHA] functional class, treatment, echocardiographic, and electrocardiogram variables), functional (cardiopulmonary exercise test parameters), sociodemographic (sex, schooling, and parents’ education), and behavioral (physical activity and motivation) characteristics.

**Results:**

A total of 100 patients (mean [SD] age, 12.7 [3.1] years; 52 boys [52.0%]) and 107 controls (mean [SD] age, 11.7 [3.3] years; 54 boys [50.5%]) were included. The V̇o_2_max was lower in patients than controls, expressed as *z* scores (mean [SD] score, −1.49 [1.48] vs −0.16 [0.97]; *P* < .001) or raw values (mean [SD] value, 32.2 [7.9] vs 40.2 [8.5] mL/kg/min; *P* < .001). Moderate to vigorous physical activity levels were lower in patients than in controls (mean [SD] level, 42.0 [23.6] vs 48.2 [20.4] min/d; *P* = .009). The final multivariable model explained 80% of the V̇o_2_max by integrating clinical (lower NYHA functional class, absence of ventricular dilatation, and absence of implantable cardioverter-defibrillator), functional (higher forced vital capacity and ventilatory anaerobic threshold), sociodemographic (male sex, normal progression of schooling, and higher maternal educational level), and behavioral (higher self-reported physical activity and motivation toward physical activity) parameters.

**Conclusions and Relevance:**

This cross-sectional study suggests that levels of cardiopulmonary fitness and physical activity were lower in children and adolescents with inherited cardiac disease than in healthy controls, even after adjusting for use of β-blockers and using modern pediatric reference models. Assessing cardiopulmonary fitness among children with inherited cardiac disease can contribute to engaging in a shared decision-making process for sports participation and preventive interventions, such as early cardiac rehabilitation programs.

## Key Points

**Question:**

What are the levels of cardiopulmonary fitness among children and adolescents with inherited cardiac disease compared with healthy controls and the factors associated with maximum oxygen uptake (V̇o_2_max) in young patients?

**Findings:**

This cross-sectional study with 207 participants showed lower levels of cardiopulmonary fitness among youths with inherited cardiac disease. The multivariable model explained 80% of V̇o_2_max, including clinical, functional, sociodemographic, and behavioral parameters.

**Meaning:**

This study suggests that assessing cardiopulmonary fitness among children with inherited cardiac disease can be incorporated into the shared decision-making process for sports participation and may help identify eligible patients for early preventive cardiac rehabilitation programs.

## Teaser

This cross-sectional study evaluates cardiopulmonary fitness and physical activity among children with inherited cardiac disease vs controls and identifies the factors associated with maximum oxygen uptake in this population.

## Introduction

Cardiopulmonary fitness is an important marker of health^1,2^ associated with quality of life among children with cardiac conditions.^3^ Cardiopulmonary exercise testing (CPET) has become a key examination in various pediatric preventive cardiology programs.^4,5^ Cardiopulmonary fitness decreases 4 times faster in children with congenital heart disease than in the healthy population,^6^ resulting in a higher risk of myocardial infarction and chronic cardiovascular disease later in life.^7,8,9^ Given these risks, a preventive approach through early cardiac rehabilitation has demonstrated benefits on cardiopulmonary fitness and quality of life for children with congenital heart disease.^4,10^

Apart from congenital heart disease, other life-threatening pediatric cardiac conditions, such as inherited arrhythmias or cardiomyopathies, have received far less attention regarding preventive interventions. However, patients with these conditions face barriers to engaging in physical activities due to parental overprotection, lack of self-efficacy, and fear of cardiac events.^11,12,13^ In addition, concerns about mortality risks associated with sports participation have led physicians to impose long-standing restrictions despite limited supporting evidence.^14,15,16^ More recent studies challenge these assumptions, instead promoting shared decision-making to empower patients with accurate information and support their autonomy in deciding whether to engage in sports.^17,18,19,20^

However, implementing shared decision-making to minimize the risk of cardiac events among children with inherited cardiac disease remains challenging. Beyond the cardiologic phenotype and genotype, a comprehensive evaluation of cardiopulmonary fitness and physical activity would enable professionals to ascertain individual exercise capacities and recommend appropriate intensities and modalities. By being fully informed of their specific fitness and physical activity levels, patients may effectively participate in the decision-making process.^18^ Previous reports on pediatric inherited cardiac disease indicated limitations in cardiopulmonary fitness and physical activity. However, these investigations lacked control groups, involved small sample sizes,^11,21,22,23,24,25,26^ and did not use contemporary and reliable pediatric CPET reference values.^27,28^

This multicenter, prospective, controlled cross-sectional study aimed to evaluate cardiopulmonary fitness and physical activity among children and adolescents with inherited cardiac disease compared with healthy controls using contemporary pediatric *z* scores. We hypothesized that these markers of physical health would be affected in this population, in association with various factors other than the underlying cardiac disease.

## Methods

### Study Design and Settings

This prospective, multicenter, controlled cross-sectional study was conducted from February 1, 2021, to June 20, 2023, with a 2-week follow-up, in 7 French pediatric university hospitals (expert centers for inherited cardiac disease). The study was conducted following the Good Clinical Practice principles and the Declaration of Helsinki^29^ after receiving ethical approval from Comité de Protection des Personnes Sud-Est-VI and preregistration at ClinicalTrials.gov (NCT04712136). Written informed consent was obtained from all parents or legal guardians, and oral assent was obtained from all children. The study design was previously published^30^ and followed the Strengthening the Reporting of Observational Studies in Epidemiology (STROBE) reporting guideline.^31^

### Participants

Children and adolescents aged 6 to 17 years who received a clinical or genetic diagnosis of an inherited cardiac arrhythmia (long QT syndrome, Brugada syndrome, or catecholaminergic polymorphic ventricular tachycardia) or an inherited cardiomyopathy (hypertrophic, dilated, or restrictive cardiomyopathy or arrhythmogenic right ventricular cardiomyopathy) were prospectively recruited during a routine outpatient cardiology consultation. Healthy controls (aged 6-17 years) were consecutively referred for CPET with nonsevere functional symptoms (eg, murmur, palpitation, or dyspnea). Participants with normal cardiologic checkup results (physical examination, electrocardiography [ECG], and echocardiography), no chronic disease (eg, pulmonary, kidney, or neurologic), and no long-term medication were eligible for inclusion in the control group. Patients whose CPET results indicated cardiologic abnormalities requiring further investigation (eg, abnormal ECG results or blood pressure) were not eligible. Participants who were not able to answer the questionnaires independently or with assistance from a clinical research associate (eg, non-French speakers) were not eligible.

### Cardiopulmonary Fitness

Cardiopulmonary fitness was evaluated using a unified pediatric cycle ergometer CPET protocol consistent with previous studies (details are provided in the eAppendix in Supplement 1).^6,27^ The main parameter reflecting cardiopulmonary fitness was the maximum oxygen uptake (V̇o_2_max), an important marker of health.^2,32^ The study primary outcome was V̇o_2_max expressed as a *z* score from the recent pediatric CPET reference equations, derived from a nonlinear model using natural logarithms of V̇o_2_max, height, and body mass index (BMI; calculated as weight in kilograms divided by height in meters squared).^27,28^

Other CPET parameters were collected, including the ventilatory anaerobic threshold (VAT) estimated using the V-slope method,^33^ ventilatory efficiency, oxygen uptake efficiency slope, and maximum oxygen pulse. One investigator per center interpreted CPET, and parameters were expressed in *z* scores from the same pediatric CPET reference equations.^27,28^ Impaired V̇o_2_max and VAT were defined as a *z* score less than −1.64, corresponding to the fifth percentile for standard normal distribution.^27,28^

For didactic purposes, as most results in previous pediatric CPET studies have been presented using raw values or historic linear reference models, V̇o_2_max and VAT were also expressed in milliliters per kilogram per minute and as a percentage of the estimated value from the weight-based equations of Cooper et al.^34^ A V̇o_2_max of less than 80% or a VAT of less than 55% indicated impaired cardiopulmonary fitness using the equations of Cooper et al.^4,34^

### Physical Activity

An accelerometer (ActiGraph GT3X; ActiGraph LLC) was placed on the child’s right hip to measure daily physical activity.^35^ Instructions were given to continuously wear it for 14 days, excluding the time spent sleeping, and intensities were classified according to thresholds of Evenson et al.^36^ Participants’ data were deemed valid if they wore the accelerometer for at least 3 days with a minimum of 10 hours per day.^37^

Children also completed 2 questionnaires under the clinical research assistant’s supervision: the Ricci and Gagnon physical activity questionnaire^38^ and the Motivation for Health-Oriented Physical Activity questionnaire.^39^ Details on physical activity assessment are provided in the eAppendix in Supplement 1.

### Other Data Collected

In both groups, the following sociodemographic characteristics, collected through a survey, and clinical data were as follows: age, sex, BMI, New York Heart Association (NYHA) functional class, 2-dimensional ECG parameters, 12-lead electrocardiographic variables, and information on patients’ family situations, parents’ employment, and child’s school curriculum. Inherited cardiac disease–related data included age at diagnosis, prior symptoms, genetics, disease severity and type, medications, pacemaker, implantable cardioverter-defibrillator, and prior cardiac surgical procedures.

### Sample Size

The Quality of Life in Children With Inherited Cardiomyopathy or Arrhythmia (QUALIMYORYTHM) trial was designed to compare health-related quality of life among children with inherited cardiac disease vs healthy children (primary outcome) and evaluate cardiopulmonary fitness and physical activity (secondary outcomes).^30^ Based on previous studies, we hypothesized to observe a mean (SD) difference in the total PedsQL (Pediatric Quality of Life Inventory) of 7 (15) points.^40,41^ With a 90% power, a 5% bilateral α risk, and potentially 10% loss to follow-up or missing data on the primary outcome, a total of 214 patients were required (107 in each group).^42,43,44^ This sample size exceeds the minimum number of 138 participants required to observe a V̇o_2_max mean (SD) relevant difference of 5.5 (9.4) mL/kg/min, using a 2-sided α risk of 5%, 90% power, and 10% attrition rate, based on previous studies.^6,21,28^

### Statistical Analysis

The study population was described with mean (SD) values for quantitative variables and with numbers and percentages for qualitative variables. Continuous variable distributions were tested using the Shapiro-Wilk test. Quantitative variables were compared using the *t* test when the distribution was Gaussian and the Mann-Whitney test otherwise. For qualitative variables, the groups were compared using the χ^2^ test or the Fisher exact test.

For comparisons of CPET parameters, a covariate analysis adjusted for β-blocker treatment, and BMI was used to account for their potential association (only for β-blocker treatment for *z* scores, as BMI is included in the model). The effect size was estimated using the absolute difference in mean values or frequencies with a 95% CI.

To identify the factors associated with V̇o_2_max *z* score among children with inherited cardiac disease, a multiple linear regression model was used. When potential factors were considered missing at random, a multiple linear imputation was implemented using a fully conditional specification method. For better stability, the number of imputations in the imputation process was 10, and all baseline characteristics were used in the imputation model. The percentage of missing data for main factors varied between 3% and 10%. All clinically relevant parameters (functional, clinical, sociodemographic, and behavioral) with *P* ≤ .20 in the univariate analysis were included in the model. Variables were selected using backward selection based on the Akaike information criterion. No collinearity between variables was detected with variance inflation factors. The normality of residues in the final model was tested using the Shapiro-Wilk test. All *P* values were from 2-sided tests, and results were deemed statistically significant at *P* < .05. Analyses were performed using SAS, version 9.04 (SAS Institute Inc).

## Results

### Population Characteristics

The total expected number of 214 participants were enrolled in the study, but 6 children with inherited cardiac disease and 1 in the control group were unable to undergo CPET (eFigure in Supplement 1). Therefore, the final analysis included 207 participants: 100 patients (mean [SD] age, 12.7 [3.1] years; 52 boys [52.0%] and 48 girls [48.0%]) and 107 controls (mean [SD] age, 11.7 [3.3] years; 54 boys [50.5%] and 53 girls [49.5%]) (Table 1). Both groups were similar in terms of sex and age; however, children with inherited cardiac disease were taller, heavier, and had a moderately higher BMI and a lower resting heart rate than healthy controls. A higher proportion of unemployed mothers was observed in the patient group, and children’s parents were more likely to be employees than higher intellectual or intermediate professionals.

**Table 1.** Population Characteristics

Characteristic	Participants, No./total No. (%)		*P* value
	Patients (n = 100)	Controls (n = 107)	
Anthropometric data			
Age range, y			
6-7	8/100 (8.0)	17/107 (15.9)	.23
8-9	9/100 (9.0)	17/107 (15.9)	
10-11	18/100 (18.0)	18/107(16.8)	
12-13	17/100 (17.0)	18/107 (16.8)	
14-15	24/100 (24.0)	18/107 (16.8)	
16-17	24/100 (24.0)	19/107 (17.8)	
Sex			
Girls	48/100 (48.0)	53/107 (49.5)	.83
Boys	52/100 (52.0)	54/107 (50.5)	
Weight, mean (SD), kg	50.6 (18.3)	44.7 (16.8)	.02
Height, mean (SD), cm	156.1 (16.8)	150.8 (18.2)	.03
BMI, mean (SD)	20.2 (4.6)	18.9 (3.5)	.05
Clinical characteristics			
Resting heart rate, mean (SD), beats/min	70.6 (15.9)	77.9 (12.3)	<.001
Resting systolic blood pressure, mean (SD), mm Hg	110.4 (14.4)	109.3 (12.1)	.88
Resting diastolic blood pressure, mean (SD), mm Hg	60.8 (11.1)	60.6 (9.0)	.56
NYHA functional class^a^			
I	83/90 (92.2)	77/79 (97.5)	.18
II	7/90 (7.8)	2/79 (2.5)	
Electrocardiography results			
QRS, mean (SD), ms	91.1 (17.5)	85.2 (9.7)	.02
QTc, mean (SD), ms	436.5 (35.7)	405.9 (28.0)	<.001
Echocardiography results			
Left ventricular ejection fraction, mean (SD), %	66.5 (9.8)	68.9 (5.7)	.10
Left ventricular diastolic diameter, mean (SD), mm	43.7 (11.1)	41.8 (8.7)	.73
Septal thickness, mean (SD), mm	11.4 (12.9)	7.6 (7.3)	<.001
Spirometry results			
FEV_1_, mean (SD), *z* score	−0.94 (1.19)	−0.25 (1.09)	<.001
FVC, mean (SD), *z* score	−1.05 (1.45)	−0.42 (1.13)	.001
FEV_1_ to FVC ratio, mean (SD), *z* score	0.50 (2.81)	0.36 (1.15)	.64
Sociodemographic data			
Patient’s educational level			
Primary school	29/99 (29.3)	38/104 (36.5)	.55
Middle school	41/99 (41.4)	40/104 (38.5)	
High school	26/99 (26.3)	25/104 (24.0)	
Dropped out of school	3/99 (3.0)	1/104 (1.0)	
Patient enrolled in special education^b^	6/92 (6.5)	3/98 (3.1)	.32
Patient’s grade progression			
Normal progression or skipped a grade	92/98 (93.9)	95/97 (97.9)	.28
Repeated a grade	6/98 (6.1)	2/97 (2.1)	
Patient’s siblings, No.			
0	5/79 (6.3)	1/78 (1.3)	.19
1-2	50/79 (63.3)	57/78 (73.1)	
≥ 3	24/79 (30.4)	20/78 (25.6)	
Mother’s educational level			
Middle school diploma or no education	30/98 (30.6)	21/101 (20.8)	.26
High school diploma	20/98 (20.4)	17/101 (16.8)	
Bachelor’s degree or associate’s degree	25/98 (25.5)	30/101 (29.7)	
Master’s degree or higher	23/98 (23.5)	33/101 (32.7)	
Mother’s professional situation			
Full time, part time, or retired	72/98 (73.5)	87/99 (87.9)	.009
Temporary work, on sick leave, or unemployed	26/98 (26.5)	12/99 (12.1)	
Mother’s type of employment			
Farmer, shopkeeper, or business owner	1/75 (1.3)	9/85 (10.6)	.001
Executive or higher intellectual profession or intermediate profession	20/75 (26.7)	41/85 (48.2)	
Employee or worker	54/75 (72.0)	35/85 (41.2)	
Father’s educational level			
Middle school diploma or no education	36/90 (40.0)	29/97 (29.9)	.11
High school diploma	21/90 (23.3)	16/97 (16.5)	
Bachelor’s degree or associate’s degree	13/90 (14.4)	25/97 (25.8)	
Master’s degree or higher	20/90 (22.2)	27/97 (27.8)	
Father’s professional situation			
Full time, part time, or retired	84/91 (92.3)	93/96 (96.9)	.20
Temporary work, on sick leave, or unemployed	7/91 (7.7)	3/96 (3.1)	
Father’s type of employment			
Farmer, shopkeeper, or business owner	14/82 (17.1)	23/88 (26.1)	.04
Executive or higher intellectual profession or intermediate profession	23/82 (28.1)	34/88 (38.6)	
Employee or worker	45/82 (54.9)	31/88 (35.2)	

Abbreviations: BMI, body mass index (calculated as weight in kilograms divided by height in meters squared); FEV_1_, forced expiratory volume in the first second; FVC, forced vital capacity; NYHA, New York Heart Association; QTc, QT interval corrected by the Bazett formulae.

^a^ None of the participants had an NYHA functional class higher than II.

^b^ Special education enrollment indicates participant enrolled in a special school to account for their particular educational status.

Among the 100 patients, 54 (54.0%) had inherited arrhythmia, and 46 (46.0%) had inherited cardiomyopathy (Table 2). A genetic variation, extracted from medical records, was found in 77 of the 85 patients (90.6%) tested. β-Blockers were prescribed for 73 patients (73.0%) and flecainide for 3 patients (3.0%), and 10 patients (10.0%) had an implantable cardioverter-defibrillator.

**Table 2.** Characteristics of Children With Inherited Cardiac Disease

Characteristic	Participants, No./total No. (%) (n = 100)
Age at diagnosis, mean (SD), y	7.5 (4.7)
Inherited arrhythmia	
Total	54/100 (54.0)
Long QT syndrome	47/54 (87.0)
Catecholaminergic polymorphic ventricular tachycardia	7/54 (13.0)
Brugada syndrome	0/54 (0)
Inherited cardiomyopathy	
Total	46/100 (46.0)
Hypertrophic cardiomyopathy	24/46 (52.2)
Dilated cardiomyopathy	19/46 (41.3)
Arrhythmogenic right ventricular cardiomyopathy	3/46 (6.5)
Genetic variants	
Total	77/85 (90.6)
Inherited arrhythmia genetic variants	
* KCNQ1*	26/77 (33.8)
* KCNH2*	12/77 (15.6)
* KCNJ2*	3/77(3.9)
* RYR2*	5/77 (6.5)
* PKP2*	2/77 (2.6)
Other	2/77 (2.6)
Inherited cardiomyopathy genetic variants	
* MYBPC3*	7/77 (9.1)
* MYH7*	10/77 (12.9)
Other	10/77 (12.9)
Cardiovascular events	
Cardiac arrest	4/100 (4.0)
Ventricular tachycardia	1/100 (1.0)
Supraventricular tachycardia	3/100 (3.0)
Syncope	5/100 (5.0)
Cardiac infections	2/100 (2.0)
Comorbidity	
Respiratory disease	5/100 (5.0)
Neurovascular disease	4/100 (4.0)
Metabolic disease	2/100 (2.0)
Urologic and digestive disease	3/100 (3.0)
Treatment	
Antiarrhythmic agents^a^	73/100 (73.0)
Heart failure treatment^b^	12/100 (12.0)
Noncardiac treatment	10/100 (10.0)
Implantable cardioverter-defibrillator	10/100 (10.0)
Pacemaker	1/100 (1.0)

^a^ β-Blockers (nadolol, atenolol, bisoprolol, carvedilol, propranolol), calcium channel inhibitors, and flecainide antiarrhythmic.

^b^ Diuretics, angiotensin-converting enzyme inhibitors, aldosterone antagonists, and glifozin.

### Results on Cardiopulmonary Fitness

The V̇o_2_max was significantly lower in the group of children with inherited cardiac disease than in healthy controls, whether expressed as mean (SD) *z* score (−1.49 [1.48] vs −0.16 [0.97]; *P* < .001), mean (SD) raw values (32.2 [7.9] vs 40.2 [8.5] mL/kg/min; *P* < .001), or mean (SD) percentage-estimated values (78.5% [18.7%] vs 99.2% [18.6%]; *P* < .001) (Table 3). Similar results were observed after adjusting for β-blocker treatment and BMI, with an adjusted absolute V̇o_2_max *z* score difference of −0.69 (95% CI, –1.21 to –0.18), representing a raw difference of –4.0 mL/kg/min (95% CI, –7.0 to –1.0 mL/kg/min). No significant difference was observed for *z* scores for V̇o_2_max and VAT between disease subgroups (eTable in Supplement 1). The VAT was lower in children with inherited cardiac disease, even after adjusting for β-blocker treatment and BMI.

**Table 3.** Cardiopulmonary Fitness Among Children With Inherited Cardiac Disease vs Healthy Controls

CPET parameters	Mean (SD)		Absolute difference (95% CI)	*P* value	Adjusted absolute difference (95% CI)^a^	Adjusted *P* value^a^
	Patients (n = 100)	Controls (n = 107)				
**Maximal CPET parameters**						
V̇o_2_max						
*z* Score	−1.49 (1.48)	−0.16 (0.97)	−1.33 (−1.68 to −0.99)	<.001	−0.69 (−1.21 to −0.18)	.008
Raw value, mL/kg/min	32.2 (7.9)	40.2 (8.5)	−8.0 (−10.2 to −5.7)	<.001	−4.0 (−7.0 to −1.0)	.01
Percentage-estimated value, %	78.5 (18.7)	99.2 (18.6)	−20.7 (−25.8 to −15.6)	<.001	−8.4 (−14.7 to −2.1)	.009
Impaired V̇o_2_max, No./total No. (%)						
*z* Score <−1.64	40/100 (40.0)	7/107 (6.5)	33.5 (22.8 to 44.1)	<.001	NA	NA
Percentage-estimated value <80%	49/100 (49.0)	14/107 (13.1)	35.9 (24.2 to 47.6)	<.001	NA	NA
Workload						
*z* Score	−1.40 (1.56)	−0.10 (1.12)	−1.30 (−1.67 to −0.92)	<.001	−0.55 (−1.10 to 0.01)	.05
Raw value, W	116.8 (49.4)	134.2 (60.3)	−17.3 (−32.4 to −2.2)	.06	−5.6 (−26.8 to 15.6)	.60
Heart rate						
*z* Score	−4.00 (3.04)	−0.21 (1.75)	−3.79 (−4.48 to −3.10)	<.001	−0.69 (−1.57 to 0.20)	.13
Raw value, beats/min	152.5 (27.1)	186.2 (15.5)	−33.7 (−39.8 to −27.6)	<.001	−6.5 (−14.4 to 1.4)	.11
Oxygen pulse						
*z* Score	0.05 (1.41)	−0.09 (1.10)	0.14 (−0.21 to 0.49)	.22	−0.46 (−0.99 to 0.07)	.09
Raw value, mL/beat	10.4 (3.6)	9.3 (3.2)	1.1 (0.1 to 2.0)	.03	−0.3 (−1.6 to 1.0)	.66
Respiratory exchange rate						
*z* Score	−0.53 (1.20)	−0.19 (1.04)	−0.34 (−0.65 to −0.04)	.007	−0.21 (−0.69 to 0.27)	.39
Raw value, ratio	1.12 (0.11)	1.15 (0.10)	−0.02 (−0.05 to 0.01)	.08	−0.01 (−0.06 to 0.03)	.54
Ventilatory efficiency						
*z* Score	0.48 (1.17)	0.51 (1.16)	−0.02 (−0.36 to 0.32)	.71	0.02 (−0.49 to 0.53)	.94
Raw value, ratio	32.2 (5.1)	32.8 (5.3)	−0.6 (−2.1 to 0.9)	.44	−0.6 (−2.9 to 1.6)	.58
Breathing reserve						
*z* Score	0.38 (1.19)	−0.11 (0.99)	0.49 (0.17 to 0.82)	.001	0.22 (−0.26 to 0.70)	.36
Raw value, %	31.9 (17.5)	24.8 (14.2)	7.2 (2.4 to 11.9)	.001	3.3 (−3.7 to 10.3)	.36
Tidal volume						
*z* Score	−1.24 (2.59)	0.09 (1.15)	−1.33 (−1.89 to −0.76)	<.001	−0.48 (−1.32 to 0.35)	.26
Raw value, mL	1249.8 (576.8)	1364.5 (555.5)	−114.7 (−271.5 to 42.1)	.13	6.9 (−211.8 to 225.6)	.95
Systolic blood pressure						
Raw value, mm Hg	138 (27)	140 (25)	−2.5 (−9.8 to 4.8)	.45	1.0 (−9.5 to 11.5)	.86
**CPET parameters at VAT**						
VAT						
*z* Score	−1.28 (1.51)	0.03 (0.99)	−1.31 (−1.67 to −0.95)	<.001	−0.57 (−1.10 to −0.04)	.03
Raw value, mL/kg/min	22.4 (7.1)	29.0 (7.4)	−6.6 (−8.6 to −4.6)	<.001	−2.7 (−5.4 to 0.0)	.05
Percentage-estimated VAT, %	54.6 (17.5)	71.5 (16.8)	−16.9 (−21.6 to −12.2)	<.001	−5.3 (−11.3 to 0.7)	.08
Impaired VAT, No./total No. (%)						
*z* Score <−1.64	31/97 (32.0)	7/107 (6.5)	25.4 (15.0 to 35.8)	<.001	NA	NA
Percentage-estimated value <55%	46/97 (47.4)	18/107 (16.8)	30.6 (18.4 to 42.8)	<.001	NA	NA
Workload						
Raw value, W	70.7 (33.5)	86.6 (42.0)	−15.9 (−26.4 to −5.4)	.005	−8.5 (−24.3 to 7.3)	.29
Heart rate						
Raw value, beats/min	123.4 (24.0)	156.5 (15.1)	−33.1 (−38.7 to −27.4)	<.001	−9.0 (−16.5 to −1.5)	.02
Oxygen pulse						
Raw value, mL/beat	8.7 (2.9)	7.9 (2.7)	0.7 (0.0 to 1.5)	.03	0.01 (−1.1 to 1.1)	.99

Abbreviations: BMI, body mass index; CPET, cardiopulmonary exercise test; NA, not applicable; VAT, ventilatory anaerobic threshold; V̇o_2_max, maximal oxygen uptake.

^a^ Adjusted for β-blocker treatment and BMI for all parameters (except for *z* scores with adjustment for β-blocker treatment only).

Overall, group differences in cardiopulmonary fitness were identified more frequently using the *z* score model than using the linear equations of Cooper et al.^34^ In addition, in both groups, fewer children were considered to have poor cardiopulmonary fitness using the *z* score model than the linear equations of Cooper et al.^34^ Among the 100 patients and 107 controls, impaired V̇o_2_max was observed in 40 patients (40.0%) and 7 healthy children (6.5%) using the *z* score model compared with 49 patients (49.0%) and 14 controls (13.1%) using the equations of Cooper et al^34^ (both *P* *<* .001) (Table 3). Similar findings were observed using the VAT threshold: 31 of 97 patients (32.0%) vs 7 of 107 controls (6.5%) had impaired VAT with the *z* score model, and 46 of 97 patients (47.4%) vs 18 of 107 controls (16.8%) had impaired VAT with the linear equations of Cooper et al^34^ (all *P* *<* .001). All CPET parameter comparisons are presented in Table 3.

### Physical Activity Results

The mean (SD) valid wear time for accelerometry was appropriate in both the patient group (10.6 [3.3] days and 13.4 [1.7] h/d) and the control group (9.5 [2.9] days and 12.9 [1.2] h/d). Seven patients and 9 controls were excluded from the analysis due to insufficient valid data (≥3 days with 10 h/d). Children with inherited cardiac disease engaged in significantly less vigorous and moderate to vigorous physical activity (vigorous: mean [SD], 12.0 [10.5] vs 16.1 [12.0] min/d; *P* = .005; moderate to vigorous: mean [SD], 42.0 [23.6] vs 48.2 [20.4] min/d; *P* = .009) and more sedentary time (mean [SD], 568.6 [122.5] vs 524.3 [101.4] min/d; *P* = .02) than healthy controls (Table 4). No significant differences were found for light and moderate intensity.^45^ On questionnaires, patients had lower scores of physical activity and intrinsic motivation than controls.

**Table 4.** Physical Activity Among Children With Inherited Cardiac Disease vs Healthy Controls

Physical activity measurement	Mean (SD)		Absolute difference (95% CI)	*P* value
	Patients (n = 93)	Controls (n = 98)		
Accelerometry-derived physical activity				
Sedentary time, min/d	568.6 (122.5)	524.3 (101.4)	44.3 (12.3 to 76.4)	.02
Light intensity, min/d	203.4 (62.9)	215.3 (61.3)	−12.0 (−29.7 to 5.8)	.12
Moderate intensity, min/d	29.9 (15.2)	31.92 (12.7)	−2.0 (−6.0 to 2.0)	.09
Vigorous intensity, min/d	12.0 (10.5)	16.1 (12.0)	−4.1 (−7.3 to −0.9)	.005
MVPA, min/d	42.0 (23.6)	48.2 (20.4)	−6.1 (−12.4 to 0.2)	.009
Steps/d	7662.3 (2991.3)	8366.8 (2315.4)	−704.5 (1471.3 to 62.3)	.02
Meeting WHO guidelines, No./total No. (%)^a^^,^^b^	14/81 (17.3)	20/87 (23.0)	−5.7 (−17.8 to 6.4)	.36
Physical activity questionnaire (total score)	24.9 (0.3)	26.9 (4.2)	−2.0 (−3.4 to −0.6)	.008
Motivation toward health-oriented physical activity				
Intrinsic motivation	5.2 (1.6)	5.7 (1.2)	−0.6 (−1.0 to −0.2)	.01
Extrinsic motivation				
Integrated regulation	4.2 (1.9)	5.1 (1.6)	−0.8 (−1.3 to −0.3)	.002
Identified regulation	5.3 (1.5)	5.8 (1.2)	−0.5 (−0.9 to −0.1)	.03
Introjected regulation	3.8 (1.6)	4.4 (1.6)	−0.7 (−1.1 to −0.2)	.007
External regulation	1.6 (1.1)	1.7 (1.2)	−0.1 (−0.4 to 0.2)	.52
Amotivation	1.6 (1.0)	1.4 (0.8)	0.2 (0.0 to 0.5)	.13

Abbreviations: MVPA, moderate to vigorous physical activity; WHO, World Health Organization.

^a^ WHO activity guidelines for children aged between 5 and 17 years are at least 60 minutes per day of moderate to vigorous intensity.^45^

^b^ Compliance with international guidelines (≥60 minutes of MVPA/d) was evaluated only for children and adolescents with at least 6 valid days.

### Factors Associated With Maximum Oxygen Uptake *z* Score Among Children and Adolescents With Inherited Cardiac Disease

Among children with inherited cardiac disease, the V̇o_2_max *z* score was associated with clinical, functional, sociodemographic, and behavioral parameters in univariate analysis (Table 5). The amounts of daily moderate to vigorous physical activity and daily vigorous physical activity assessed by accelerometers were lower for patients with inherited heart disease but were not associated with V̇o_2_max in the multivariable model (univariate *r* ≤ 0.30). In multivariable analysis, the factors associated with a higher V̇o_2_max *z* score were the absence of an implantable cardioverter-defibrillator, lower NYHA functional class, greater QRS duration, lower left ventricular diastolic diameter, higher forced vital capacity, higher VAT, male sex, normal progression of schooling, having no siblings, higher educational level for mother, lower educational level for father, higher score on the physical activity questionnaire, and lower extrinsic motivation regulated externally. The final multivariable model explained 80% of the V̇o_2_max *z* score.

**Table 5.** Factors Associated With Maximum Oxygen Uptake *z* Score Among Children With Inherited Cardiac Disease

Parameter	Univariate analysis			Multivariable analysis (n = 100)			
	Correlation coefficient *r* or mean (SD) value	Raw β (95% CI)	*P* value	Adjusted β (95% CI)	Standardized β	*P* value
Clinical parameters						
Resting systolic blood pressure, mm H*g*^a^	*r* = 0.15	0.02 (−0.01 to 0.04)	.15	NA	NA	NA
Mean QRS, ms^a^	*r* = 0.14	0.02 (0.00 to 0.04)	.08	0.02 (0.01 to 0.03)	0.22	<.001
Left ventricular diastolic diameter, mm^a^	*r* = 0.27	0.03 (0.00 to 0.05)	.04	−0.02 (−0.03 to −0.01)	−0.17	.01
Type of disease						
Inherited arrhythmia	−1.60 (1.43)	0.25 (−0.34 to 0.84)	.41	NA	NA	NA
Inherited cardiomyopathy	−1.35 (1.56)	NA	NA	NA	NA	NA
NYHA functional class^a^^,^^b^						
I	−1.35 (1.33)	2.15 (1.05 to 3.25)	<.001	0.79 (0.20 to 1.38)	0.15	.009
II	−3.50 (2.17)	NA	NA	NA	NA	NA
β-Blockers^a^						
No	−0.85 (1.46)	0.88 (0.23 to 1.52)	.01	NA	NA	NA
Yes	−1.73 (1.43)	NA	NA	NA	NA	NA
Implantable cardioverter-defibrillator^a^						
No	−1.36 (1.43)	1.26 (0.31 to 2.21)	.01	0.78 (0.24 to 1.31)	0.16	.005
Yes	−2.62 (1.53)	NA	NA	NA	NA	NA
Functional parameters						
FEV_1_ *z* score^a^	*r* = 0.36	0.45 (0.20 to 0.71)	<.001	NA	NA	NA
FVC *z* score^a^	*r* = 0.39	0.41 (0.20 to 0.61)	<.001	0.23 (0.13 to 0.33)	0.25	<.001
VAT *z* score^a^	*r* = 0.74	0.72 (0.59 to 0.85)	<.001	0.58 (0.47 to 0.68)	0.62	<.001
Sociodemographic parameters						
Sex^a^						
Boys	−1.24 (1.34)	0.53 (−0.06 to 1.11)	.08	0.43 (0.12 to 0.74)	0.15	.006
Girls	−1.76 (1.59)	NA	NA	NA	NA	NA
Patient’s grade progression^a^						
Normal progression or skipped a grade	−1.42 (1.48)	0.93 (−0.30 to 2.17)	.14	NA	NA	NA
Repeated a grade	−2.35 (1.43)	NA	NA	NA	NA	NA
Patient’s special education^a^^,^^c^						
No	−1.38 (1.38)	1.66 (0.44 to 2.87)	.01	0.58 (0.00 to 1.17)	0.11	.05
Yes	−3.04 (2.34)	NA	NA	NA	NA	NA
Mother’s educational level^a^						
Master’s degree or higher	−0.89 (1.23)	0.85 (0.12 to 1.59)	.07	0.53 (0.11 to 0.96)	0.15	.05
Bachelor’s degree	−1.57 (1.31)	0.17 (−0.55 to 0.88)	NA	0.05 (−0.33 to 0.44)	0.02	NA
Middle school, high school diploma, or no education	−1.74 (1.63)	NA	NA	NA	NA	NA
Mother’s professional situation^a^						
Full time, part time, or retired	−1.35 (1.60)	0.46 (−0.21 to 1.14)	.17	NA	NA	NA
Temporary work, on sick leave, or unemployed	−1.82 (1.10)	NA	NA	NA	NA	NA
Mother’s type of employment^a^						
Farmer, shopkeeper, or business owner	−1.66 (0.00)	−0.02 (−3.11 to 3.08)	.06	NA	NA	NA
Executive or higher intellectual profession or intermediate profession	−0.67 (1.27)	0.97 (0.17 to 1.77)	NA	NA	NA	NA
Employee or worker	−1.64 (1.63)	NA	NA	NA	NA	NA
Father’s educational level^a^						
Master’s degree or higher	−0.91 (1.26)	0.58 (−0.18 to 1.34)	.15	−0.34 (−0.77 to 0.09)	−0.10	0.01
Bachelor’s degree	−1.90 (0.99)	−0.40 (−1.30 to 0.50)	NA	−0.73 (−1.21 to −0.26)	−0.18	NA
Middle school, high school diploma, or no education	−1.50 (1.62)	NA	NA	NA	NA	NA
No. of patient’s siblings^a^						
≥3	−2.49 (1.40)	−1.31 (−2.75 to 0.13)	.01	−0.86 (−1.39 to −0.34)	−0.26	.005
1-2	−1.35 (1.49)	−0.17 (−1.54 to 1.20)	NA	−0.69 (−1.16 to −0.22)	−0.23	NA
0	−1.18 (1.60)	NA	NA	NA	NA	NA
Behavioral parameters						
Physical activity questionnaire total score^a^	*r* = 0.36	0.08 (0.03 to 0.13)	.003	0.03 (0.01 to 0.06)	0.14	.009
Intrinsic motivation^a^	*r* = 0.43	0.44 (0.28 to 0.61)	<.001	NA	NA	NA
Extrinsic motivation						
Identified regulation^a^	*r* = 0.34	0.38 (0.20 to 0.56)	<.001	NA	NA	NA
Introjected regulation^a^	*r* = 0.12	0.16 (−0.02 to 0.34)	.09	NA	NA	NA
External regulation^a^	*r* = −0.21	−0.22 (−0.50 to 0.06)	.13	−0.24 (−0.37 to −0.10)	−0.17	.001
Vigorous intensity, min/d^d^	*r* = 0.26	0.04 (0.00 to 0.06)	.01	NA	NA	NA
MVPA, min/d^a^	*r* = 0.30	0.02 (0.00 to 0.03)	.01	NA	NA	NA
Meeting WHO guidelines^e^						
Yes	−0.94 (1.33)	0.63 (−0.22 to 1.48)	.14	NA	NA	NA
No	−1.58 (1.48)	NA	NA	NA	NA	NA

Abbreviations: FEV_1_, forced expiratory volume in the first second; FVC, forced vital capacity; MVPA, moderate to vigorous physical activity; NA, not applicable; NYHA, New York Heart Association; VAT, ventilatory anaerobic threshold; WHO, World Health Organization.

^a^ Variables included in the multivariable analysis.

^b^ None of the participants had an NYHA functional class higher than II.

^c^ Special education enrollment indicates participant enrolled in a special school to account for their particular education status.

^d^ There was a strong correlation between vigorous physical activity and MVPA (*r* = 0.87), which led us to include only MVPA in the final model.

^e^ WHO activity guidelines for children aged between 5 and 17 years are a mean of 60 minutes per day of moderate to vigorous intensity.

## Discussion

From a cohort of 207 children, this multicenter, prospective, controlled cross-sectional study found that children with inherited cardiac disease had lower levels of cardiopulmonary fitness than healthy controls, using the new pediatric CPET reference *z* scores,^27,28^ and lower physical activity levels. Overall, the clinical, functional, sociodemographic, and behavioral parameters used in the multivariable model could explain 80% of V̇o_2_max.

The V̇o_2_max was significantly lower in children with inherited cardiac disease than in healthy controls, even after adjusting for use of β-blockers, with an absolute *z* score difference of −0.69, representing a magnitude of the difference of 4.0 mL/kg/min. These results are important considering that a V̇o_2_max decrease above 3.5 mL/kg/min (ie, 1 metabolic equivalent of task) is associated with a higher risk of mortality among adults.^46,47^ Furthermore, reduced maximal workload can suggest muscular alterations (eg, fiber type, mitochondrial density, and muscle strength) in patients with inherited cardiac diseases.^21^

The reduced cardiopulmonary fitness level in children with inherited cardiac disease, which is an important marker of health,^2^ may lead to higher cardiovascular risk factors and comorbidities later in life.^32,48^ These findings are concerning in light of established associations between reduced cardiopulmonary fitness and poor psychosocial health,^49^ particularly among these youths with inherited cardiac diseases in which psychosocial well-being may already be compromised.^50,51^

Our results align with several previous findings^11,21,26,52^ but offer stronger evidence from the study design, use of standardized CPET procedures, and analysis using contemporary *z* score pediatric reference values. The use of historical linear models for reference equations has been previously criticized, as the association between body weight and oxygen consumption is not strictly linear, particularly for individuals with extreme weight.^53,54^ In response, new reference *z* score equations based on a natural logarithmic model can enhance the interpretability of individual variation with reference to normative data, while accounting for population variability in demographic factors (eg, weight and height).^27,28^ These *z* score equations can also be valuable in the follow-up of patients; however, future longitudinal studies are necessary to determine which specific thresholds or changes in V̇o_2_max *z* score over time have prognostic implications for young individuals with inherited cardiac disease.

The clinical, functional, sociodemographic, and behavioral parameters used in the multivariable model explained 80% of V̇o_2_max in children with inherited heart disease. Cardiopulmonary fitness depends on respiratory, cardiac, and muscle functions, as well as anthropometric and behavioral parameters. Among the children with inherited heart disease, severe cardiac conditions (high NYHA functional class, left ventricle dilatation, or implantable cardioverter-defibrillator) were associated with a lower V̇o_2_max. β-Blocker use was not associated with V̇o_2_max, which aligns with previous findings in congenital heart disease.^6^ One explanation is that β-blocker use may also improve diastolic function and compensate for chronotropic incompetence by increasing stroke volume, as in cardiomyopathy and long QT syndrome.^21,55^ The V̇o_2_max was also associated with functional parameters, such as forced vital capacity and oxygen consumption at VAT, suggesting, respectively, restrictive lung patterns or impaired muscular fitness^6,56^ as we observed in children with congenital heart disease^6^ and long QT syndrome.^21^

This study also highlighted the association between sociodemographic parameters and cardiopulmonary fitness among children with inherited cardiac disease. Sex, normal progression of schooling, having no siblings, and parents’ level of education were associated with V̇o_2_max. The lower V̇o_2_max associated with female sex can be due to body composition and blood hemoglobin concentration.^57^ In addition, early sex discrimination, such as sports restrictions imposed at the time of diagnosis yet not captured in this study, could also help explain this finding.^37^ Higher maternal educational level was associated with higher V̇o_2_max_,_ which aligns with previous findings in the general population.^58^

In this study, the amounts of daily moderate to vigorous physical activity and daily vigorous physical activity assessed by accelerometers were lower among patients with inherited heart disease but were not associated with V̇o_2_max in the multivariable model (univariate *r* ≤ 0.30), whereas a higher score on the physical activity questionnaire was associated with a higher V̇o_2_max in the multivariable analysis. These findings may stem from the use of a nonvalidated questionnaire, potentially leading to an overestimation of physical activity levels.^59^ In addition, hereditary factors may determine up to 50% of V̇o_2_max, thereby limiting the effect of physical activity.^60^ Last, guidelines for inherited cardiac disease recommend avoiding vigorous intensity,^18,19,20^ which is one of the more likely intensities to increase V̇o_2_max.^61,62^ Therefore, cardiopulmonary fitness and physical activity should be considered complementary but distinct health-related factors among children with inherited cardiac disease.

Motivation toward physical activity was also associated with V̇o_2_max. According to the self-determination theory,^63^ higher extrinsic motivation externally regulated in these children (eg, running to obtain a higher grade in physical education class) may be a barrier to autonomously and enjoyably engaging in physically active behaviors in the long term,^11^ potentially resulting in lower V̇o_2_max.

In this study, more than one-third of children with inherited cardiac disease had poor cardiopulmonary fitness (40.0% with impaired V̇o_2_max), making them potentially eligible for an exercise training program. To promote physical activity, we recommend shared decision-making, taking into account the clinical, functional, sociodemographic, and behavioral profile of the child and his or her family. A multidisciplinary approach involving pediatric cardiologists, specialist nurses, and exercise physiologists could help determine the most appropriate strategy to promote physical activity while limiting life-threatening events.^17^ Early preventive hybrid cardiac rehabilitation programs can open new avenues for the management of youths with inherited cardiac disease, as shown in congenital heart disease.^4^

### Limitations

This study had several limitations. Other unmeasured variables, such as hereditary components, diet, lean body mass, weather, and school time could be associated with cardiopulmonary fitness and physical activity. The discrepancy between objective and subjective assessments of physical activity warrants further investigation, starting with exhaustive validation of a pediatric physical activity questionnaire.

Although a recent study observed good compliance with current guidelines among children with inherited cardiac disease,^64^ physicians’ and parents’ behaviors regarding exercise restrictions were not evaluated in this study. The heterogeneity of subgroups of patients with inherited cardiac disease restricted the analysis of conditions, such as arrhythmogenic right ventricular cardiomyopathy, that likely lead to greater sports restrictions.

## Conclusions

In this cross-sectional study, the levels cardiopulmonary fitness and physical activity were lower among children with inherited cardiac disease than in healthy controls, even after adjusting for use of β-blockers. Using contemporary and reliable pediatric CPET reference values strengthened our results and can be introduced for the follow-up of these patients over time. The clinical, functional, sociodemographic, and behavioral parameters were the multidimensional parameters associated with V̇o_2_max. Assessment of cardiopulmonary fitness among children with inherited cardiac disease would enable professionals to ascertain individual exercise capacities and identify suitable candidates for early cardiac rehabilitation programs by providing the appropriate exercise intensity and modality.

## Notes

Supplement 1. **eAppendix.** Additional Study Assessment
eReferences.
**eFigure.** Flow Chart of Participants Included in the Analysis **eTable.** Comparison of Z-Score VO_2max_ and VAT Across Subgroups of Patients and Controls

Supplement 2. **Nonauthor Collaborators.** Quality of Life in Children With Inherited Cardiomyopathy or Arrhythmia (QUALIMYORYTHM) Study Group members

Supplement 3.
Data Sharing Statement
