# Supplementary material for: Cardiopulmonary Fitness and Physical Activity Among Children and Adolescents With Inherited Cardiac Disease
Source: JAMA Netw Open. 2025 Feb 25;8(2):e2461795. doi: 10.1001/jamanetworkopen.2024.61795 (PMC11862974; doi:10.1001/jamanetworkopen.2024.61795)
Supplement: Supplement 1. — eAppendix. Additional Study Assessment eReferences. eFigure. Flow Chart of Participants Included in the Analysis eTable. Comparison of Z-Score VO2max and VAT Across Subgroups of Patients and Controls [file jamanetwopen-e2461795-s001.pdf]

Supplementary Online Content

Souilla L, Werner O, Huguet H, et al; Quality of Life in Children With Inherited Cardiomyopathy or Arrhythmia (QUALIMYORYTHM) Study Group. Cardiopulmonary fitness and physical activity among children and adolescents with inherited cardiac disease. *JAMA Netw Open.* 2025;8(2):e2461795. doi:10.1001/jamanetworkopen.2024.61795

eAppendix. Additional Study Assessment
eReferences.
eFigure. Flow Chart of Participants Included in the Analysis
eTable. Comparison of Z-Score VO_{2max} and VAT Across Subgroups of Patients and Controls

This supplementary material has been provided by the authors to give readers additional information about their work.

© 2025 Souilla L et al. *JAMA Network Open.*

eAppendix. Additional Study Assessment

1. CPET procedures and quality control

Cardiopulmonary fitness was evaluated using a unified pediatric CPET protocol consistent with our previous similar studies.[1–3] Exercise laboratories for each center followed a high-quality score rating (≥10), according to ATS/ACCP statement on CPET,[4] employing adapted equipment and procedures: adapted pediatric face masks, calibration of gas analyzers, utilization of breath-to-breath measurement software, 12-lead electrocardiogram equipment, and a pulse oximeter, mask leak assessment before and during exercise with monitoring of VO_2/W ratio. Spirometry using a common gas device was systematically performed before the exercise test with a flow-volume curve and measurement of forced expiratory volume in 1s (FEV1), forced vital capacity (FVC), and the FEV1/FVC ratio (FEV1%), with normalization to theoretical values.[5]
Children in both groups underwent a cycle ergometer to obtain a homogeneous incremental overall duration between 8 and 12 minutes, including a) 1-minute baseline b) 3-minute warm-up (10-20 watts) c) fixed increments of 10-20 watts/min (3.5 W/kg in adolescent boys and 3 W/kg in girls and preadolescent boys); d) pedaling rate of 60-80 revolutions/min; e) 3-minute active recovery; f) 2-minute rest. CPET was considered maximal when one of the following four criteria was reached: (1) respiratory exchange ratio ≥1, (2) maximum heart rate >85% of maximal age-predicted heart rate (220-age), (3) limit of the patient's tolerance despite verbal encouragement, and (4) plateau of VO_2 despite increasing exercise intensity.
Upon compilation of data from all centers, most participants met at least two of the CPET maximal criteria, except for two patients (2.0%) and one control (0.5%).

2. Details on physical activity assessment

Data from the accelerometer were processed using a sampling rate of 30 Hz, an epoch of 15 s, and the non-wearing period was calculated using Choi's algorithm.[6] Intensities were classified using the Evenson threshold: sedentary (0-100 counts/min), light physical activity (101-2295 counts/min), moderate physical activity (2296-4011 counts/min), vigorous physical activity (≥4012 counts/min), and moderate-to-vigorous physical activity (MVPA ≥2296 counts/min).[7]

The physical activity questionnaire Ricci-Gagnon,[8] previously used in pediatric cardiology,[9] includes nine items, and scoring was performed if all items were collected: one for sedentary behaviors, four for sports and leisure activities, and four for daily life activities.

The motivation for health-oriented physical activity (EMAPS) questionnaire[10] used includes an 18-item divided into three domains and was scored if all items within each domain were present: intrinsic motivation, observed when individuals adopt a behavior for the pleasure and satisfaction they derive from it; extrinsic motivation, observed when behavior is a means of achieving a positive result or preventing a negative one; and amotivation, observed when individuals feel that there is no way for them to obtain positive results from their actions.[10,11]

© 2025 Souilla L et al. *JAMA Network Open.*

eReferences.

1. Amedro P, Matecki S, Pereira dos Santos T, et al. Reference Values of Cardiopulmonary Exercise Test Parameters in the Contemporary Paediatric Population. *Sports Medicine - Open*. 2023;9(1):68. doi:10.1186/s40798-023-00622-3

2. Gavotto A, Mura T, Rhodes J, et al. Reference values of aerobic fitness in the contemporary paediatric population. *European Journal of Preventive Cardiology*. 2023;30(9):820-829. doi:10.1093/eurjpc/zwad054

3. Souilla L, Avesani M, Boisson A, et al. Cardiorespiratory fitness, muscle fitness, and physical activity in children with long QT syndrome: A prospective controlled study. *Frontiers in Cardiovascular Medicine*. 2023;9. Accessed March 7, 2023. https://www.frontiersin.org/articles/10.3389/fcvm.2022.1081106

4. American Thoracic Society, American College of Chest Physicians. ATS/ACCP Statement on cardiopulmonary exercise testing. *Am J Respir Crit Care Med*. 2003;167(2):211-277. doi:10.1164/rccm.167.2.211

5. Quanjer PH, Stanojevic S, Cole TJ, et al. Multi-ethnic reference values for spirometry for the 3–95-yr age range: the global lung function 2012 equations. *European Respiratory Journal*. 2012;40(6):1324-1343. doi:10.1183/09031936.00080312

6. Choi L, Liu Z, Matthews CE, Buchowski MS. Validation of accelerometer wear and nonwear time classification algorithm. *Med Sci Sports Exerc*. 2011;43(2):357-364. doi:10.1249/MSS.0b013e3181ed61a3

7. Evenson KR, Catellier DJ, Gill K, Ondrak KS, McMurray RG. Calibration of two objective measures of physical activity for children. *J Sports Sci*. 2008;26(14):1557-1565. doi:10.1080/02640410802334196

8. Vuillemin A, Denis G, Guillemin F, Jeandel C. [A review of evaluation questionnaires for physical activity]. *Rev Epidemiol Sante Publique*. 1998;46(1):49-55.

9. Amedro P, Gavotto A, Huguet H, et al. Early hybrid cardiac rehabilitation in congenital heart disease: the QUALIREHAB trial. *European Heart Journal*. Published online March 2, 2024:ehae085. doi:10.1093/eurheartj/ehae085

10. Boiché J, Gourlan M, Trouilloud D, Sarrazin P. Development and validation of the ‘Echelle de Motivation envers l’Activité Physique en contexte de Santé’: A motivation scale towards health-oriented physical activity in French. *J Health Psychol*. 2019;24(3):386-396. doi:10.1177/1359105316676626

11. Deci EL, Ryan RM. “Facilitating optimal motivation and psychological well-being across life’s domains”: Correction to Deci and Ryan (2008). *Canadian Psychology / Psychologie canadienne*. 2008;49(3):262-262. doi:10.1037/0708-5591.49.3.262

© 2025 Souilla L et al. *JAMA Network Open.*

eFigure. Flow Chart of Participants Included in the Analysis

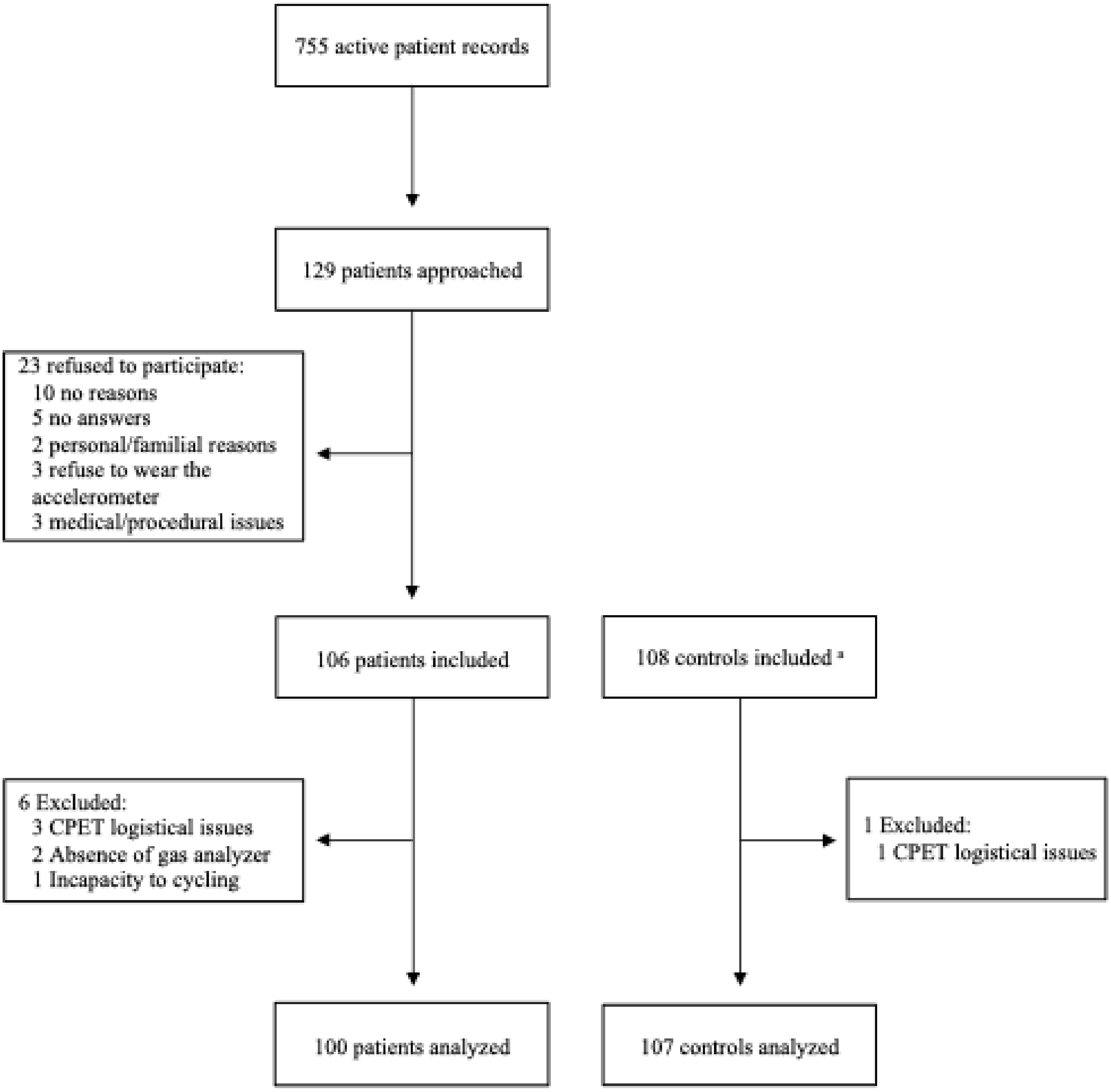

Footnotes: [a] Healthy children and adolescents were recruited upon voluntary participation following normal clinical check-up.

Abbreviations: CPET, cardiopulmonary exercise test.

eTable. Comparison of Z-Score VO_{2max} and VAT Across Subgroups of Patients and Controls

CPET Parameters	Control [(a)] *(N=107)*	Long QT syndrome [(b)] *(N=47)*	Hypertrophic cardiomyopathy [(c)] *(N=24)*	Dilated cardiomyopathy [(d)] *(N=18)*	ARVC + CPVT [(e)] *(N=10)*
VO_{2max} Z-score, Mean (SD)	-0.16 (±0.97) [b, c, e]	-1.49 (±1.47) [a]	-1.55 (±1.54) [a]	-1.11 (±1.62)	-1.95 (±1.24) [a]
VO_2 at VAT Z-score, Mean (SD)	0.03 (±0.99) [b, c, e]	-1.33 (±1.45) [a]	-1.36 (±1.36) [a]	-0.70 (±1.64)	-1.61 (±1.63) [a]

Footnotes: Superscript letters indicate a significant difference ($p<0.05$) with the group. For pairwise comparisons, false discovery rate correction was applied. No significant difference was found between patients' subgroups.

Abbreviations: ARVC, Arrhythmogenic right ventricular cardiomyopathy; CPET, cardiopulmonary exercise test; CPVT, Catecholaminergic polymorphic ventricular tachycardia; VAT, ventilatory anaerobic threshold; VO_{2max}, maximal oxygen uptake.

© 2025 Souilla L et al. *JAMA Network Open.*
