# Supplementary material for: Cardiopulmonary Fitness and Physical Activity Among Children and Adolescents With Inherited Cardiac Disease
Source: JAMA Netw Open. 2025 Feb 25;8(2):e2461795. doi: 10.1001/jamanetworkopen.2024.61795 (PMC11862974; doi:10.1001/jamanetworkopen.2024.61795)
Supplement: Supplement 2. — Nonauthor Collaborators. Quality of Life in Children With Inherited Cardiomyopathy or Arrhythmia (QUALIMYORYTHM) Study Group members [file jamanetwopen-e2461795-s002.pdf]

Supplemental Online Content: Nonauthor Collaborators
*First name, last name, and suffix (if applicable) are required and will appear in PubMed.

*Group Name(s): Quality of Life in Children With Inherited Cardiomyopathy or Arrhythmia (QUALIMYORYTHM) Study Group							
***First Name and Middle Initial(s)**	***Last Name**	***Suffix (eg, Jr, III)**	Academic Degrees	Institution	Location (city, state/province, country)	Role or Contribution, eg, chair, principal investigator	Group (if more than 1 Group listed in the byline) and/or Subgroup (eg, Steering Committee)
Hamouda	Abassi		PhD	Montpellier University Hospital	Montpellier, France	clinical research associate	
Aymeric	Boisson		MD	Montpellier University Hospital	Montpellier, France	co-investigator	
Anne	Requirand		MSc	Montpellier University Hospital	Montpellier, France	CPET lab technician	
Annie	Auer		RN	Institut Saint-Pierre	Palavas-Les-Flots, France	CPET lab technician	
Vincent	Probst		MD,PhD	Nantes University Hospital	Nantes, France	co-investigator	
Solène	Prigent		MD	Nantes University Hospital	Nantes, France	co-investigator	
Quentin	Hauet		MD	Nantes University Hospital	Nantes, France	co-investigator	
Christelle	Haddad		MD	Hospices Civils de Lyon	Lyon, France	co-investigator	
Claire	Bertail-Galoin		MD	Lyon University Hospital	Lyon, France	co-investigator	
Xavier	Iriart		MD	Bordeaux University Hospital	Bordeaux, France	co-investigator	
Julie	Thomas-Chabaneix		MD	Bordeaux University Hospital	Bordeaux, France	co-investigator	
Pierre-Marie	Duboue		MD	Bordeaux University Hospital	Bordeaux, France	co-investigator	
Amandine	Ruissel		MSc	Bordeaux University Hospital	Bordeaux, France	clinical research associate	
Jean-Bernard	Selly		MD	La Réunion University Hospital	La Réunion, France	co-investigator	
