# Supplementary material for: Cardiopulmonary Fitness and Physical Activity Among Children and Adolescents With Inherited Cardiac Disease
Source: JAMA Netw Open. 2025 Feb 25;8(2):e2461795. doi: 10.1001/jamanetworkopen.2024.61795 (PMC11862974; doi:10.1001/jamanetworkopen.2024.61795)
Supplement: Supplement 3. — Data Sharing Statement [file jamanetwopen-e2461795-s003.pdf]

Data Sharing Statement

Souilla. Cardiopulmonary Fitness and Physical Activity Among Children and Adolescents With Inherited Cardiac Disease. *JAMA Netw Open*. Published February 25, 2025.
doi:10.1001/jamanetworkopen.2024.61795

Data
Data available: Yes
Data types: Deidentified participant data
How to access data: mc-picot@chu-montpellier.fr
When available: With publication

Supporting Documents
Document types: Statistical/analytic code, Informed consent form
How to access documents: mc-picot@chu-montpellier.fr
When available: With publication

Additional Information
Who can access the data: researchers whose proposed use of the data has been approved
Types of analyses: for any purpose
Mechanisms of data availability: with a signed data access agreement
